# Human mesenchymal stromal cells in adhesion to cell‐derived extracellular matrix and titanium: Comparative kinome profile analysis

**DOI:** 10.1002/jcp.27116

**Published:** 2018-07-30

**Authors:** Marta Baroncelli, Gwenny M. Fuhler, Jeroen van de Peppel, Willian F. Zambuzzi, Johannes P. van Leeuwen, Bram C. J. van der Eerden, Maikel P. Peppelenbosch

**Affiliations:** ^1^ Department of Internal Medicine, Erasmus MC University Medical Center Rotterdam Rotterdam The Netherlands; ^2^ Department of Gastroenterology and Hepatology, Erasmus MC University Medical Center Rotterdam Rotterdam The Netherlands; ^3^ Laboratorio de Bioensaios e Dinâmica Celular, Departamento de Quimica e Bioquimica Instituto de Biociências, Universidade Estadual Paulista‐UNESP São Paulo Brazil

**Keywords:** cell adhesion, extracellular matrix, kinome profiling, osteoblasts, titanium

## Abstract

The extracellular matrix (ECM) physically supports cells and influences stem cell behaviour, modulating kinase‐mediated signalling cascades. Cell‐derived ECMs have emerged in bone regeneration as they reproduce physiological tissue‐architecture and ameliorate mesenchymal stromal cell (MSC) properties. Titanium scaffolds show good mechanical properties, facilitate cell adhesion, and have been routinely used for bone tissue engineering (BTE). We analyzed the kinomic signature of human MSCs in adhesion to an osteopromotive osteoblast‐derived ECM, and compared it to MSCs on titanium. PamChip kinase‐array analysis revealed 63 phosphorylated peptides on ECM and 59 on titanium, with MSCs on ECM exhibiting significantly higher kinase activity than on titanium. MSCs on the two substrates showed overlapping kinome profiles, with activation of similar signalling pathways (FAK, ERK, and PI3K signalling). Inhibition of PI3K signalling in cells significantly reduced adhesion to ECM and increased the number of nonadherent cells on both substrates. In summary, this study comprehensively characterized the kinase activity in MSCs on cell‐derived ECM and titanium, highlighting the role of PI3K signalling in kinomic changes regulating osteoblast viability and adhesion. Kinome profile analysis represents a powerful tool to select pathways to better understand cell behaviour. Osteoblast‐derived ECM could be further investigated as titanium scaffold‐coating to improve BTE.

## 1 INTRODUCTION

The extracellular matrix (ECM) is an essential structural component present in every tissue, which physically supports cells, but also actively modulates their behaviour, by regulating the availability of bioactive molecules and by transducing mechanical signalling (Discher, Mooney, & Zandstra, 2009; Guilak et al., 2009; Hynes, 2009; Reilly & Engler, 2010). Bone matrix is composed of collagen and noncollagenous proteins to maintain bone flexibility, whereas its stiffness is achieved by hydroxyapatite crystals, which makes bone a peculiar type of connective tissue (Alford & Hankenson, 2006; Gentili & Cancedda, 2009). Because of its composition, bone ECM is essential for the structure and the strength of the bone and it also actively participates in bone formation and bone metabolism, by regulating mineralization and modulating growth factor availability (Alford & Hankenson, 2006; Bonewald & Dallas, 1994). The physical cues of bone ECM proteins are mechanosensed by bone cells via integrin‐mediated signalling, which converts the biomechanical properties of the ECM, eventually acting on cell adhesion, proliferation and differentiation (Grzesik & Robey, 1994; Hidalgo‐Bastida & Cartmell, 2010; Marie, Haÿ, & Saidak, 2014). The molecular details of the signalling pathways that mediate the relay of information from integrin engagement to altered cellular physiology remain, however, largely obscure.

In recent years, the interest in bone ECM for regenerative purposes has grown rapidly. Bone tissue engineering (BTE) applications have been proposed as bone graft substitutes in large bone defects when bone healing capacity is lost. BTE involves the combination of scaffolds (osteoconduction), osteogenic factors (osteoinduction), and autologous mesenchymal stromal cells (MSCs; osteogenesis) to mimic the native bone ECM structure and stimulate the osteogenic differentiation of local progenitors, driving new bone formation (Gemini‐Piperni, Takamori et al., 2014; Meijer, de Bruijn, Koole, & van Blitterswijk, 2007). Scaffolds serve as a structural template for osteogenesis, being biocompatible and osteoconductive (Bose, Roy, & Bandyopadhyay, 2012). Among the several scaffolds that can be used for BTE, titanium shows good mechanical properties and it can be tailored in porosity to suit cell adhesion. Although not bioresorbable, titanium scaffolds are already clinically used for orthopaedic and dental implants and in load‐bearing areas for their good mechanical properties (Holland & Mikos, 2006). As scaffolds do not reproduce the native structure of bone ECM, decellularized ECMs represent an alternative cell‐instructive microenvironment to guide endogenous repair (Badylak, Freytes, & Gilbert, 2009; Benders et al., 2013). In this context, cell‐secreted ECMs have also been proposed, as they are readily available and can be customized for the use as scaffold‐coating (Decaris, Binder, Soicher, Bhat, & Leach, 2012; Fitzpatrick & McDevitt, 2015; Hoshiba, Lu, Kawazoe, & Chen, 2010). We and others demonstrated that osteoblast‐derived ECM stimulates MSC osteogenesis and promotes bone formation (Baroncelli M., 2018; Datta, Holtorf, Sikavitsas, Jansen, & Mikos, 2005; Mauney, Kaplan, & Volloch, 2004). Moreover, cell‐secreted ECMs have already been used to coat and modify titanium surfaces, showing that ECM influences gene expression and enhances osteogenic differentiation of MSCs (Datta, et al., 2005; Q. P. Pham, et al., 2008; M. T. Pham, Reuther, & Maitz, 2003). In this context, the aim of this study was to compare the naturally secreted devitalized ECM to titanium and investigate how they differentially regulate cell adhesion.

Kinase activity lies at the core of cell signal transduction, as activation of specific kinases mediates the induction of signalling cascades resulting into cellular processes such as cell metabolism, differentiation, and cytoskeletal rearrangements during cell adhesion (Peppelenbosch, Frijns, & Fuhler, 2016; Robertson, et al., 2015; Zaidel‐Bar & Geiger, 2010; Zambuzzi, Coelho, Alves, & Granjeiro, 2011). Integrin‐mediated activation of kinase signalling cascades such as FAK and Src family kinase converts mechanical forces into biochemical signals and results in the efficient adhesion of the cell to the surface. At the same time, deregulation of kinase‐mediated signalling pathways leads to pathological states, emphasizing that studying kinase activity is crucial to understand biological functions.

The aim of our study was to assess specific kinomic changes upon MSC adhesion to cell‐derived ECM and titanium surfaces, by using tyrosine‐kinase PamChip^®^ (PamGene International BV, 's‐Hertogenbosch, The Netherland) array which to the best of our knowledge has not been used before to investigate cell adhesion of human MSCs.

## 2 MATERIALS AND METHODS

### 2.1 Cell culture and ECM preparations

Human bone marrow‐derived MSCs were used to prepare the osteopromotive devitalized ECM as previously described (Baroncelli et al., 2017). Briefly, MSCs (5,128 viable cells/cm^2^; PT‐2501; Lonza, Walkersville, MD) from a single donor at passage seven were cultured in growth medium for 2 days (α‐Mem phenol‐red free [Gibco, Paisley, UK], 10% foetal bovine serum), and osteogenically differentiated for 11 days (culture medium supplemented with 100 nM of dexamethasone and 10 mM of β glycerophosphate [Sigma‐Aldrich, St. Louis, MO] to induce the deposition of the ECM. MSCs were devitalized by freeze–thaw cycles, DNAse treatment (10 U/ml; Sigma‐Aldrich), extensive washings with phosphate buffer saline (PBS; Gibco), and sterile air drying. Devitalized ECMs were stored at −20°C until further use.

### 2.2 Tyrosine‐kinase activity profiling using PamChip peptide microarray

To check the effect of the devitalized ECM and titanium on MSC behaviour, MSCs (28,300 viable cells/cm^2^) were cultured on these surfaces in growth medium. After 4 hr, cells were scraped in M‐PER mammalian protein extraction buffer (ThermoFisher Scientific, Rockford, IL) containing halt phosphatase and protease inhibitors (ThermoFisher Scientific), allowed to lyse at 4°C for 10 min and lysates were cleared by centrifugation at 14,000*g* for 10 min. Supernatants were stored at −80°C until use. Cell lysates (5 μg protein for all samples) were loaded on a PamChip tyrosine‐kinase microarray (PamGene International BV, ’s‐Hertogenbosch, The Netherlands). PamChip^®^ is a high‐throughput and cost‐effective peptide array that allows the study of kinome profile changes without a priori assumptions (Peppelenbosch, 2012). In the PamChip platform, cell lysates are continuously pumped past 144 consensus peptide‐sequences spotted on a three‐dimensional porous microarray, and the phosphorylation of their specific target substrates by kinases present in the whole cell lysate is fluorescently detected, describing the entire tyrosine‐kinase activity profile within a single experiment (Diks et al., 2004; Lemeer et al., 2007; Sikkema et al., 2009). Phosphorylation of the 144 kinase substrates on the array was detected by using FITC‐labelled secondary antibody. After array washing, images were taken every 5 min to create real‐time kinetics data. Signal intensities of the three technical replicates for each substrate were quantified using Bionavigator software (version 6.1.42.1; PamGene International BV). A complete list of phosphopeptides on PamChip is depicted in Supporting Information Table 1. The internal positive control peptide ART_003_EAI(pY)AAPFAKKKXC was not considered for further analysis. Kinase reactions start at *t* = 640 s. Subsequently, kinase reactions for different peptides show markedly different kinetics. Most peptides act according to classical biochemical theory, with the derivative of the initial reaction speed approximating maximal velocity (*V*_max_) for phosphorylation of this peptides. For data analysis of these peptides *V*_max_ was established by calculating the tangent of apparent peptide phosphorylation between 640 and 1,040 s and were classified as early *V*_max_ peptides (Supporting Information Figure 1a). Phosphorylation of other peptides showed considerable lag time, followed by a quick rise in speed of phosphorylation and subsequent decay according to conventional biochemical theory, yielding sigmoid curves of substrate phosphorylation when plotted against the time domain. For peptides which upon visual inspection displayed such a Maxwell–Boltzmann‐like activation kinetics, *V*_max_ was calculated by determining the tangent of substrate phosphorylation between 1,040 and 1,440 s and were classified as mid *V*_max_ (Supporting Information Figure 1b). Finally, a group of peptides displayed very slow initial activation followed by a rapid increase in reaction velocity toward the end of the experiment. These peptides were identified by inspection of the visual aspect of the curve and classified as late *V*_max_, whereas *V*_max_ was calculated by using the tangent of apparent substrate phosphorylation between 1,440 and 1,840 s (Supporting Information Figure 1c). A detailed flowchart of kinome profile analysis is presented in Supporting Information Figure 1d. *V*_max_ values below zero were artificially set to zero. Only *V*_max_ values with average above zero were considered for further analysis. Markov state analysis was performed to determine “on” and “off” calls of peptide phosphorylation on ECM and titanium (Alves et al., 2015). In detail, for each substrate the 143 peptides were ranked for *V*_max_ intensity, and a linear trend line was set for the lowest 60 peptides considered as background. Peptides whose average phosphorylation minus 1.95 times the standard deviation being higher than the background signal were considered as Markov‐positive “on” calls and further analyzed (Supporting Information Figure 2a,b).

### 2.3 Kinome array analysis

Protein and gene annotations of the kinase substrates on PamChip were searched through Uniprot Knowledgebase (www.uniprot.org). Markov‐positive peptides were analyzed through Qiagen’s Ingenuity^®^ Pathway Analysis (IPA^®^; Qiagen, Redwood City CA; www.qiagen.com/ingenuity) against human genome provided by Ingenuity Knowledge Base as background. Gene IDs of the parent proteins of Markov‐positive peptides on ECM and titanium were used in IPA. As the peptide ART_004_EAIYAAPFAKKKXC is phosphorylated by ABL1 kinase if artificial (Kua et al., 2012) as in our case, ABL1 was also included in the IPA analysis. Consensus phosphopeptides representing different phosphorylation sites of the same protein were considered together. The Canonical Pathway analysis tool was used for IPA analysis. Intracellular signalling pathways not restricted to a specific cellular type were selected and used to generate the heat map using R, together with the relative Gene IDs of the phosphorylated kinase substrates.

Because specific kinases activate signalling cascades, the kinase substrates that were phosphorylated in cells on ECM and on titanium were further fitted into signalling pathways and cell‐related functions as previously described (Sikkema et al., 2009) to confirm IPA analysis.

### 2.4 Immunoblot analysis

Some of the activated kinases revealed by PamChip array were validated by western blot analysis as described (with modifications; Fuhler et al., 2009; Queiroz et al., 2012). Briefly, cell lysates (40 µg) were prepared as for kinome profile analysis, mixed with 2× Laemmli buffer, separated by sodium dodecyl sulfate‐polyacrylamide gel electrophoresis, transferred onto nitrocellulose membrane (Immobilon FL membrane; Merck KGaA, Darmstadt, Germany) and nonspecifically blocked with Odyssey buffer (LI‐COR Biosciences, Lincoln, NE). Membranes were incubated overnight with primary antibodies against pFAK (Y925; rabbit polyclonal; Signalway Antibody, College Park, MD), pERK, pPKB, pEGFR, and pSMAD1/5/8 (Cell Signaling Technology, Beverly, MA) and β‐actin loading control (mouse monoclonal; Clone sc‐47778; Santa Cruz Biotechnology, Dallas, TX). All antibodies were used 1:1,000. Membranes were probed with secondary antibody conjugated with goat‐anti‐mouse‐Alexa Fluor 680 and goat‐anti‐rabbit IRDye 800CW at 1:5,000 (LI‐COR Biosciences). Odyssey infra‐red imaging (LI‐COR Biosciences) was used to detect proteins. Quantification was performed using Odyssey 3.0 Software, LI‐COR, Lincoln, NE.

### 2.5 Cell viability analysis

To assess the effect of PI3K signalling inhibition on cell viability, PI3K signalling was inhibited by Wortmannin or LY294002 (Okada, Sakuma, Fukui, Hazeki, & Ui, 1994). MSCs were cultured on ECM for 4 hr in growth culture medium in the presence of 10 μM of Wortmannin (in dimethyl sulfoxide [DMSO]; Sigma), 10 μM of LY294002 (in DMSO; Cayman Chemical, Ann Arbor, MI) or vehicle control. After 4 hr, MSCs were gently rinsed with PBS and both floating and adherent cells were collected in Laemmli buffer.

To confirm that Wortmannin and LY294002 effectively inhibited PI3K signalling, cell lysates treated with and without PI3K inhibitors were probed for the presence of pPKB and pERK (p42/44) by immunoblot analysis.

To investigate if the PI3K inhibitors affected cell viability, the presence of poly‐ADP‐ribose polymerase (PARP) and caspase 3 and its relative cleaved forms were detected by western blot analysis (antibodies from Cell Signaling Technology; Somasundaram et al., 2013).

### 2.6 Cell adhesion analysis

To check the effect of PI3K signalling inhibition on cell adhesion, MSCs were cultured in osteogenic conditions on cell‐derived ECM, in the presence of increasing concentrations of Wortmannin and LY294002 (0.1, 1, and 10 μM in DMSO for both). MSCs in adhesion to titanium with and without the highest concentration of Wortmannin and LY294002 (10 μM in DMSO) were used for the same purpose. MSCs with vehicle were considered as control (1/200 vol/vol). After 4 hr, both floating cells and cells adhering to the substrates were collected and the fraction of adhering cells was quantified by Flow Cytometry (Accuri C6 Flow Cytometer; BD Biosciences, San Jose, CA), using counting beads (Liquid Counting Beads, BD Biosciences).

### 2.7 Statistical analysis

Area under curve (AUC) of the kinetic reaction was calculated for each Markov‐positive peptide, and nonparametric Wilcoxon matched‐pairs signed rank test used to calculate significance.

Functional attachment data were representative of three independent experiments, with one or two technical replicates per each experiment, and all values were displayed as average ± standard deviation of biological replicates otherwise indicated elsewhere. One‐way analysis of variance, followed by Bonferroni post hoc test was used to calculate significance, unless otherwise indicated.

## 3 RESULTS

### 3.1 PamChip array showed similar tyrosine‐kinase activity profiles of MSCs on ECM and on titanium

**Figure 1:**
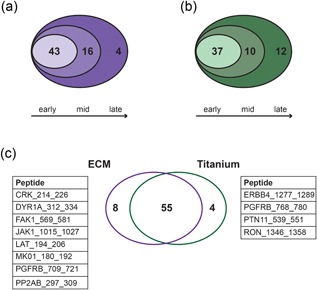
Kinome profiling of MSCs cultured on cell‐derived ECM and on titanium. (a) A total of 63 peptides on PamChip were phosphorylated by kinases in MSCs on ECM over time: 43 as early peptides, 16 as mid peptides, and 4 as late peptides. Only Markov‐positive peptides are considered. (b) A total of 59 peptides were phosphorylated on titanium over time: 37 peptides were detected since early stages, 10 were phosphorylated at mid stages, and 12 at late stages. Only Markov‐positive peptides are considered. (c) Venn diagram showing the number of peptides phosphorylated on both ECM and titanium (55), uniquely phosphorylated on ECM (8), and uniquely phosphorylated on titanium (4). Unique peptides are indicated in the tables. ECM: extracellular matrix; MSC: mesenchymal stromal cell [Color figure can be viewed at wileyonlinelibrary.com]

To investigate how surfaces influence cell behaviour, we cultured MSCs for 4 hr on either an osteopromotive osteoblast‐derived ECM or on titanium and analyzed the kinome profiles by a high‐throughput tyrosine‐kinase PamChip microarray system. Maximal velocity (*V*_max_) as the slope of phosphorylation kinetics was used as measure of peptide phosphorylation and depending on the kinetic behaviour observed, peptides were categorized as either early *V*_max_, mid *V*_max_, or late *V*_max_ (see Supporting Information Figure 1a–c for examples). Following Markov state analysis, lysates obtained from MSCs cultured on ECM yielded significant phosphorylation of 43 early *V*_max_ peptides, 16 mid *V*_max_ peptides, and 4 late *V*_max_ peptides (Figure 1a, Supporting Information Figure 2a). Thus, a total of 63 “on calls” were detected over time on ECM and considered for further analysis (Figure 1a; complete list in Table 1).

**Table 1:** List of 63 peptides phosphorylated on ECM and 59 phosphopeptides on titanium at early, mid, and late stage

Peptide #	ECM			Titanium		
	Early	Mid	Late	Early	Mid	Late
1	ART_004_EAIYAAPFAKKKXC			ART_004_EAIYAAPFAKKKXC		
2	CD79A_181_193			CD79A_181_193		
3	CDK2_8_20			CDK2_8_20		
4	CDK7_157_169			CDK7_157_169		
5	DCX_109_121			DCX_109_121		
6	EFS_246_258			EFS_246_258		
7	ENOG_37_49			ENOG_37_49		
8	EPHA1_774_786			EPHA1_774_786		
9	EPHA2_765_777			EPHA2_765_777		
10	EPHB1_921_933			EPHB1_921_933		
11	FER_707_719			FER_707_719		
12	FES_706_718			FES_706_718		
13	FRK_380_392			FRK_380_392		
14	K2C6B_53_65			K2C6B_53_65		
15	MBP_198_210			MBP_198_210		
16	MK10_216_228			MK10_216_228		
17	NCF1_313_325			NCF1_313_325		
18	NTRK2_696_708			NTRK2_696_708		
19	P85A_600_612			P85A_600_612		
20	PAXI_111_123			PAXI_111_123		
21	PAXI_24_36			PAXI_24_36		
22	PDPK1_2_14			PDPK1_2_14		
23	PECA1_706_718			PECA1_706_718		
24	PGFRB_572_584			PGFRB_572_584		
25	PLCG1_764_776			PLCG1_764_776		
26	RAF1_332_344			RAF1_332_344		
27	RB_804_816			RB_804_816		
28	RET_1022_1034			RET_1022_1034		
29	SRC8_CHICK_476_488			SRC8_CHICK_476_488		
30	SRC8_CHICK_492_504			SRC8_CHICK_492_504		
31	TYRO3_679_691			TYRO3_679_691		
32	VGFR2_944_956			VGFR2_944_956		
33	VGFR2_989_1001			VGFR2_989_1001		
34	41_654_666				41_654_666	
35	EPHA7_607_619				EPHA7_607_619	
36	JAK2_563_577				JAK2_563_577	
37	LAT_249_261				LAT_249_261	
38	PDPK1_369_381				PDPK1_369_381	
39	VGFR1_1326_1338					VGFR1_1326_1338
40	DYR1A_312_324					
41	MK01_180_192					
42	PGFRB_709_721					
43	PP2AB_297_309					
44		FAK2_572_584		FAK2_572_584		
45		PRRX2_202_214		PRRX2_202_214		
46		RASA1_453_465		RASA1_453_465		
47		EPHB1_771_783			EPHB1_771_783	
48		LCK_387_399			LCK_387_399	
49		MET_1227_1239			MET_1227_1239	
50		ANXA1_14_26				ANXA1_14_26
51		EGFR_1165_1177				EGFR_1165_1177
52		EPOR_361_373				EPOR_361_373
53		EPOR_419_431				EPOR_419_431
54		PGFRB_1002_1014				PGFRB_1002_1014
55		PGFRB_1014_1028				PGFRB_1014_1028
56		PGFRB_771_783				PGFRB_771_783
57		ZAP70_485_497				ZAP70_485_497
58		FAK1_569_581				
59		LAT_194_206				
60			TEC_512_524		TEC_512_524	
61			FGFR3_753_765			FGFR3_753_765
62			CRK_214_226			
63			JAK1_1015_1027			
64				PTN11_539_551		
65					RON_1346_1358	
66						ERBB4_1277_1289
67						PGFRB_768_780

*Note*. Peptides are in alphabetical order; numbers indicate the position of the first and last amino acid of the peptide in the complete human protein.

ECM: extracellular matrix.

On titanium, 37 peptides were phosphorylated with maximum reaction speed early in the analysis, with an additional 10 at mid stage and 12 at later stages (Supporting Information Figure 2b), yielding a total of 59 Markov‐positive peptides phosphorylated on titanium over time (Figure 1b; complete list in Table 1). Scatter plots showing the correlation of peptides at early, mid, and late stage in MSCs on ECM and on titanium are shown in Supporting Information Figure 2c,d. On both surfaces, the correlation is stronger between mid and late peptides.

Figure 1c shows the number of phosphorylated peptides on ECM and on titanium. Despite a similar number of peptides being phosphorylated on the two substrates, cultures on ECM induced a significantly higher overall phosphorylation compared to titanium (AUC of 34.1 ± 7.6 vs. 27.6 ± 6.5, *P* < 0.0001, data not shown). Several of the peptides that were significantly phosphorylated early in MSCs cultured on ECM, only achieved significant phosphorylation at later time points when cells were cultured on titanium, suggesting lower levels of active kinase present in these latter lysates. Comparing the kinase activity profiles, most of the phosphorylated peptides (55) were shared between the two substrates, highlighting a substantial overlap between the kinome profiles of MSCs in ECM and titanium, whereas eight kinase substrates were exclusively phosphorylated on the cell‐derived ECM and four on titanium (Figure 1c).

### 3.2 PamChip array revealed activation of PI3K/AKT signalling pathway

**Figure 2:**
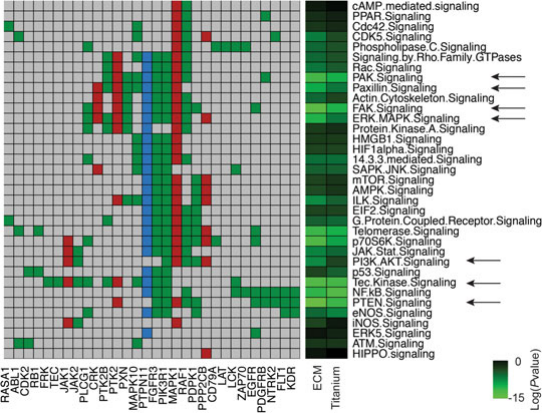
Comparative kinomes of MSCs on ECM and titanium. Heat map of the 30 gene IDs of the parent proteins of Markov‐positive phosphopeptides on ECM and titanium (bottom) involved in 35 selected intracellular signalling pathways (right) in IPA. Parent proteins of the Markov‐positive peptides are indicated as gene IDs (bottom). Green: activated in both substrates; red: uniquely activated in ECM; blue: uniquely activated on titanium; grey: none. Colour scale bar represents log (*p* value) of enrichment. Black arrows indicate signalling pathways highlighted in the text. ECM: extracellular matrix; IPA: Ingenuity® pathway analysis; MSC: mesenchymal stromal cell [Color figure can be viewed at wileyonlinelibrary.com]

We analyzed the identified activated kinase‐mediated signalling cascades by IPA, to unravel meaningful signalling changes upon cell adhesion. The 63 kinase substrates phosphorylated on ECM and the 59 on titanium were involved in many signalling pathways (complete list in Supporting Information Table 2 for ECM and Supporting Information Table 3 for titanium). We further focused on intracellular signallings, not specific for a selected cell type, resulting in a total of 30 parent gene IDs of the Markov‐positive peptides on ECM and on titanium, involved in 35 selected intracellular signalling pathways, as shown in the heat map in Figure 2. For ECM, the most induced kinases (11) were involved in phosphatase and tensin homologue (PTEN) signalling (*p* = 5.01 × 10^−15^), but also Tec kinase signalling (*p* = 3.16 × 10^−13^). Signalling cascades activated upon integrin activation such as FAK, PAK, Paxillin, and ILK signalling pathways were also activated (*p* = 3.98 × 10^−14^, 2.51 × 10^−12^, 3.09 × 10^−10^, 2.39 × 10^−8^, respectively). Of the activated kinases, 10 are identified in IPA as regulating ERK/MAPK signalling, and seven modulate PI3K/AKT signalling (*p* = 5.01 × 10^−11^ and 2.08 × 10^−8^, respectively). Moreover, Mitogen‐activated protein kinase 1 (MAPK1; peptide MK01_180_192), phosphorylated only on ECM, was involved in most of the signalling pathways, together with phosphatidylinositol 3‐kinase regulatory subunit α (PI3KR1; peptide P85A_600_612) and fibroblast growth factor receptor 3 (FGFR3; peptide FGFR3_753_765). These last kinase substrates were phosphorylated also on titanium, together with tyrosine‐protein phosphatase nonreceptor type 11 (PTPN11; peptide PTN11_539_551), which was uniquely phosphorylated in MSCs on titanium and involved in most of the activated signalling pathways (Figure 2). Most of the kinases activated on titanium were involved in nuclear factor κ‐light‐chain‐enhancer of activated B cells (NF‐κB) signalling (*p* = 2.51 × 10^−13^), Tec kinase signalling (*p* = 5.01 × 10^−12^), and PTEN signalling (*p* = 6.30 × 10^−12^). PAK, FAK, Paxillin, and ILK signalling pathways were activated on titanium as on the ECM (*p* = 7.94 × 10^−11^, 6.30 × 10^−11^, 2.29 × 10^−17^, and 5.75 × 10^−6^, respectively), as well as ERK/MAPK (*p* = 3.46 × 10^−7^) and PI3K/AKT (*p* = 1.91 × 10^−4^), confirming the overlap between these substrates.

IPA analysis revealed that the activated kinases were involved in multiple signalling cascades. In addition, we used the results of the peptide array to fit each kinase that phosphorylates a selected peptide into one specific signalling pathway, as previously done (Sikkema et al., 2009), in a more biased approach but more osteoblast‐oriented (complete list in Supporting Information Table 4). This approach confirmed the IPA analysis, as of the 63 kinase substrates phosphorylated on ECM, four induced the activation of FAK signalling and a total of 11 phosphopeptides were involved in cytoskeletal functions (Supporting Information Figure 3a,b; Supporting Information Table 5). Three peptides were clustered in MAPK signalling and three in PI3K signalling, illustrating that different approaches in kinase clustering lead to similar conclusions. Similar findings were found by clustering the 59 kinase substrates phosphorylated on titanium (Supporting Information Table 6). Phosphorylation of four peptides induce the activation of FAK signalling. Moreover, MAPK (two peptides) and PI3K (three peptides) signalling were activated also in cells in adhesion to titanium (Supporting Information Figure 3c,d).

**Figure 3:**
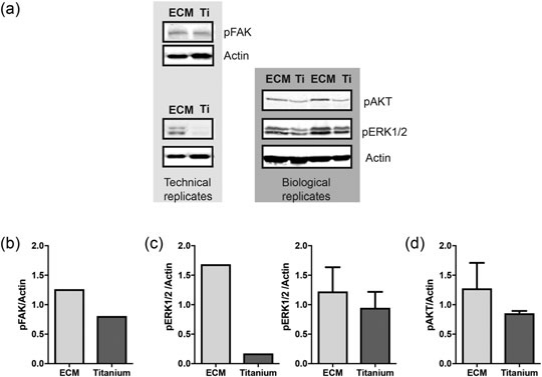
Immunoblot analysis of phosphoproteins confirmed the activation of specific intracellular signalling pathways revealed by PamChip. (a) Western blot analysis of pFAK, pERK, and pAKT in technical and biological replicates. β‐Actin was used as loading control. (b–d) Quantification of immunoblot band intensities of the selected phosphorylated kinases over β‐actin of technical and biological replicates. Bars represent average ± standard deviation. ECM: extracellular matrix; Ti: titanium

The activation of some signalling pathways on ECM and on titanium revealed by PamChip array was validated by immunoblot analysis, both in technical and biological replicates of cell lysates (Figure 3a). Overall and in line with the PamChip analyses, ECM tend to induce a higher kinase activity compared with titanium, as signalling pathways such as FAK, ERK/MAPK, and PI3K/AKT pathways were more active in cells on ECM than on titanium, as shown in Figure 3b–d by the quantification of the induced kinase relative to the loading control in technical and biological replicates. This highlights the importance of the peptide array as high‐throughput screening technique to select candidate pathways.

**Figure 4:**
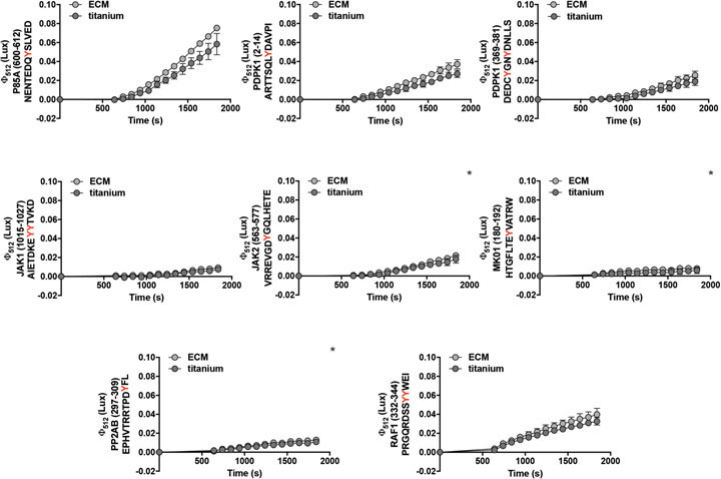
Temporal phosphorylation kinetics of selected peptides involved in PI3K/AKT signalling pathway as annotated by IPA. Peptide IDs and peptide sequences are displayed on *Y* axis; numbers indicate first and last amino acid of the complete human protein or the peptide sequence based on Uniprot annotation. Phosphotyrosines are indicated in red. Peptides that were Markov‐positive uniquely for cell lysates on ECM are indicated with *. Data represents average ± standard deviation of technical replicates. ECM: extracellular matrix; IPA: Ingenuity® pathway analysis [Color figure can be viewed at wileyonlinelibrary.com]

Quantification of kinase substrate phosphorylation in the peptide array (Supporting Information Figure 4a–c) followed the same trend as the quantification of the putative kinases of each signalling pathway by western blot (Figure 3b–d). The activation of signalling pathways revealed by IPA was the result of the phosphorylation of multiple kinase substrates (Supporting Information Table 7). For instance, the phosphorylation of eight peptides in PamChip revealed the activation of PI3K/AKT signalling in cells on ECM (Figure 4; Supporting Information Figure 4c), which was higher than on titanium (four Markov‐positive peptides), confirming the higher phosphorylation of pPKB on ECM compared with titanium (Figure 3a,d).

### 3.3 Functional consequences of reduced PI3K activation

**Figure 5:**
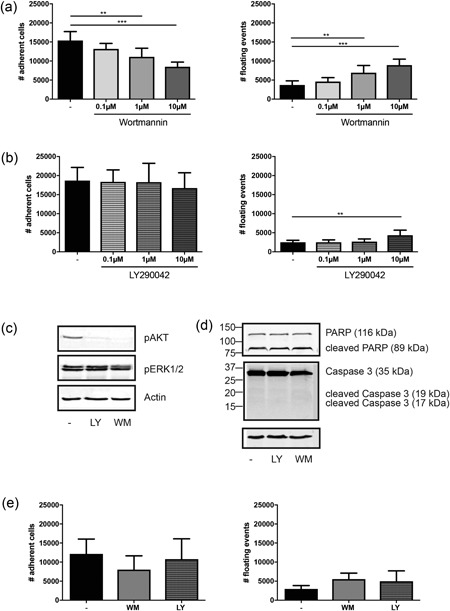
Functional effects of PI3K signalling inhibition. (a) Number of cells in adhesion to ECM (left) for 4 hr with Wortmannin 0.1, 1, and 10 μM and number of events floating in culture medium (right) in same culture conditions. (b) Quantification of MSCs in adhesion to ECM with increasing concentrations of LY294002 (left) and floating in culture medium (right). (c) Immunoblot of pAKT and pERK1/2 to confirm PI3K signalling inhibition by Wortmannin 10 μM and LY294002 10 μM in MSCs on ECM. (d) Immunoblot validation of PARP, caspase 3, and the relative cleaved forms in extracts of MSCs on ECM without and with PI3K inhibitors Wortmannin 10 μM and LY294002 10 μM. (e) Number of cells adherent to titanium (left) and nonadherent (right) after 4 hr of culture with Wortmannin 10 μM and LY294002 10 μM (left). Bars indicate average ± standard deviation of multiple independent experiments (*N* = 3; **p < 0.01; ***p < 0.001; ‐no inhibitors). ECM: extracellular matrix; LY: LY294002; MSC: mesenchymal stromal cell; WM: Wortmannin

PamChip microarray analysis revealed that the PI3K/AKT signalling pathway among others was activated in cells adhering to both substrates (Figure 2), but with a higher activity on ECM than on titanium (Figure 3a,d). The temporal kinetics of the peptides clustered in PI3K/AKT signalling are displayed in Figure 4. PI3K signalling has been shown to be important for several cellular functions, including cell adhesion. We validated this by allowing MSCs to adhere to ECM for 4 hr in the absence or presence of the PI3K kinase inhibitors Wortmannin or LY290042. Figure 5a shows that Wormannin significantly reduced cell attachment to ECM in a dose‐dependent manner (*p* < 0.001 for 10 μM of Wortmannin), by decreasing the number of cells in adhesion to the ECM (Figure 5a, left) and increasing the number of floating cells in culture medium (Figure 5a, right). Similarly, LY294002 increased the number of nonadherent cells (Figure 5b), albeit less efficiently. This is most likely due to residual PI3K activity, as shown in Figure 5c: both Wortmannin and LY294002 selectively inhibit PI3K signalling (as indicated by decreased phosphorylation of its downstream target, PKB) at a concentration of 10 μM, but Wortmannin showed a more prominent inhibitory effect. To confirm that the increase in nonadherent cells in response to PI3K inhibition was not due to induction of apoptosis by these compounds, MSCs on ECM were treated for 4 hr with and without PI3K inhibitors (Wortmannin 10 μM and LY294002 10 μM), and cleaving of PARP and caspase 3 was measured by immunoblot analysis as a hallmark of apoptosis. The presence of cleaved caspase 3 was not detected in samples treated with and without PI3K inhibitors, nor were cleaved PARP levels increased, confirming that the PI3K inhibitors were not toxic (Figure 5d).

The phosphorylation of selected kinases such as PKB (as well as ERK) was also confirmed to be reduced in cells cultured on titanium compared to cultures on ECM, also when assessing nonadherent cells (Supporting Information Figure 5a,b). Thus, we next investigated adhesion of MSCs to titanium and showed that while the highest concentration of PI3K inhibitors (10 μM) reduced adhesion, this effect did not reach statistical significance, in accordance with the lesser PI3K activation in response to titanium engagement of MSCs (Figure 5e). Overall, our data confirm the importance of PI3K signalling in cell adhesion, and suggest that kinome activity differences observed are reflected by functional consequences in MSCs.

## 4 DISCUSSION

In this study, we comprehensively described the kinome profiles of human MSCs during adherence to a cell‐derived ECM and to titanium, by successfully using PamChip array technology. MSCs on the two substrates showed a substantial overlap of kinase signatures. Cells on ECM typically activate kinase reactions that conform classical kinetics, that is that maximum reaction speeds are seen early in the experiment, and have higher level of active kinases. Importantly, without a priori assumptions, we used the PamChip kinase array to identify PI3K signalling and further functional experiments showed its importance in MSC viability and adhesion. This observation may guide rational design of novel scaffolds for tissue engineering.

Cell‐surface interplay has been studied to develop biomaterials to improve BTE (Gemini‐Piperni, Takamori, et al., 2014; Zambuzzi et al., 2011). Upon cell adhesion to a surface, mechanical forces are converted into biochemical signals by integrins, that induce the activation of FAK, Src family kinases, and an intricate network of signalling pathways, such as PI3K, MAPK ERK1/2, PKC, and Rho‐family GTPase, that eventually modulate cell behaviour (Marie et al., 2014). Osteoblast adhesion is controlled mainly by PKA, PKC, and RhoA proteins that promote cell cycle arrest and mediate cytoskeletal rearrangements (Zambuzzi, Bruni‐Cardoso, et al., 2009). In line with this, we showed that pathways such as FAK, PAK, Paxillin, ILK, and Rho GTPase family signalling were activated upon MSC adhesion to ECM and titanium. Moreover, PamChip kinase array revealed the activation of ERK/MAPK signalling. MAPKs are a central hub in controlling bone homoeostasis, as they are activated by extracellular stimuli and ECM‐mediated integrin activation via Src/FAK signalling network, but also promote osteoblast survival and differentiation by controlling osteogenic transcription factors (Greenblatt, Shim, & Glimcher, 2013; Marie et al., 2014). Cell adhesion to an ECM has been studied through mass spectrometry showing the high level of tyrosine phosphorylation in adhesion complexes, thus revealing the importance of kinases in cell adhesion (Robertson et al., 2015; Zaidel‐Bar & Geiger, 2010) The behaviour of calvarial osteoblasts has been analyzed through PepChip kinase‐array screening technology. Milani et al. (2010) studied calvarial osteoblasts in adhesion to polystyrene and reported not only the induction of FAK, Src, PKA, and PKC, but also kinases not directly related to cell adhesion such as GSK3β and Rap1A. The activation of PKA, PKC, VEGF, and adducin‐1 (ADD1) was reported in calvarial osteoblast adhesion to hydroxyapatite (Gemini‐Piperni, Milani, et al., 2014). Recently, Marumoto et al. (2017) used the PepChip platform to study the interplay between ECM and osteoblasts, showing that Hedgehog signalling regulates morphological changes in calvarial osteoblasts during a 10‐day culture on Matrigel™, confirming some of the findings by Chaves Neto et al. (2011) who investigated the osteogenic differentiation on polystyrene. In our study, PamChip kinase array was used to investigate how changes in the kinomic signature regulate human MSC adhesion to diverse substrates, such as an osteoblast‐derived ECM and titanium. PamChip contains a lower number of kinase substrates than PepChip, but allows kinetic measurements with strong reproducibility and giving quantification of end‐point signals as well as temporal kinetics of the reaction (Baharani, Trost, Kusalik, & Napper, 2017). Our PamChip results showed a big overlap between the distributions of phosphorylation in cells on the two surfaces, but also a delay in phosphorylation kinetics in cells cultured on titanium compared with MSCs on ECM, highlighting the importance of analyzing temporal kinetics.

PamChip is a cost‐effective high‐throughput array that can simultaneously identify rapid changes in kinome profiles (Peppelenbosch, 2012). PamChip and other kinase‐array platforms drive hypothesis formation, due to the variable number of putative upstream kinases that could phosphorylate the peptides. They represent powerful tools to select pathways that might be crucial in physiological functions, but the kinase activation needs to be validated by immunoblot analysis (Arsenault, Griebel, & Napper, 2011; Sikkema et al., 2009). We used IPA for functional clustering of the peptides into signalling pathways, as previously done (Kuijjer et al., 2014), and we confirmed it by fitting each peptide in one specific signalling pathway. However, software to fit phosphopeptides into cascade signalling networks needs to be implemented with kinomic‐oriented tools.

In this study, PamChip revealed the activation of PI3K/AKT in MSCs adhering to ECM and titanium, which corroborates with previous findings on polystyrene and on hydroxyapatite (Gemini‐Piperni et al., 2014; Milani et al., 2010). Conversely, PI3K/AKT signalling was found to be downregulated during osteogenic differentiation in standard culture conditions and on Matrigel (Chaves Neto et al., 2011; Marumoto et al., 2017). The PI3K/AKT signalling pathway regulates many cellular functions such as proliferation, adhesion and migration, and its activation promotes cell survival (Manning & Toker, 2017). In osteoblasts, PI3K was shown to mediate BMP2 induction of osteogenic differentiation (Baker, Sohn, & Tuan, 2015; Ghosh‐Choudhury et al., 2002; McGonnellGrigoriadis, Lam, Price, & Sunters, 2012; Mukherjee & Rotwein, 2009) and to interact with RUNX2 in controlling osteoblast and chondrocyte differentiation and migration (Fujita et al., 2004), though findings are still controversial as for the influence of PI3K signalling in osteogenic differentiation (Kratchmarova, Blagoev, Haack‐Sorensen, Kassem, & Mann, 2005; Viñals, López‐Rovira, Rosa, & Ventura, 2002). PI3K signalling is involved in integrin‐mediated signal transduction during cell adhesion (Chen, Appeddu, Isoda, & Guan, 1996; King, Mattaliano, Chan, Tsichlis, & Brugge, 1997). In this study, we showed that PI3K is involved in osteoblast adhesion to ECM and titanium, in agreement to previous studies where PI3K‐mediated AKT activity was shown to be reduced when PI3K inhibitors were present in COS7 cells and MSCs in adhesion to fibronectin (Chaudhary et al., 2000; Liu et al., 2010). When PI3K inhibitors were used, the number of nonadherent cells increased, with a stronger effect on ECM than titanium. This is probably due to the fact that PI3K signalling was more active on ECM than on titanium, as shown by the temporal kinetics of the PI3K kinase substrates, thus making it easier to visualize the inhibitory effect and highlighting the importance of performing kinetic analyses.

The mechanisms of cell adhesion to titanium has been previously investigated by using FAK and Src phosphorylation as biomarkers to monitor cell/biomaterial interplay, as FAK and Src have been proven to be phosphorylated upon integrin activation in cells when adhering to different substrates (Zambuzzi et al., 2014, 2009; Zambuzzi, Milani, & Teti, 2010). Our study using the PamChip kinase array showed FAK signalling activation upon adhesion to titanium, confirming these previous findings. Further studies are needed to investigate how the cell‐derived ECM as coating for titanium scaffolds would influence the kinome profile and osteoblast adhesion, to implement the use of ECM and titanium in BTE applications.

In summary, with this study we used a multiplex peptide array technology to assess global tyrosine kinase changes upon cell adhesion, showing that osteoblasts adhering to ECM exhibited a similar kinase signature compared with titanium, but with higher levels of active kinases present in MSCs on ECM. We successfully used PamChip kinase substrate platform to comprehensively study rapid changes in the phosphoproteomes of MSCs, and to investigate specific pathways, highlighting the importance of PI3K signalling in osteoblast viability and adhesion. We thus contributed to disentangle kinomic changes upon cell adhesion to different substrates, to develop biomaterials to improve BTE applications.

## AUTHORS’ CONTRIBUTIONS

All authors designed research. M.B., G.F., and W.Z. performed research. M.B., G.F., J.P., J.L., M.P., and B.E. analyzed data and drafted manuscript. All authors revised the final version of manuscript and have approved the final article.

## CONFLICTS OF INTEREST

All authors declare that there are no conflicts of interest.

## Supporting information

Supporting information

Supporting information

Supporting information

Supporting information

Supporting information

Supporting information

Supporting information
