# Supplementary figures and images for: Human mesenchymal stromal cells in adhesion to cell‐derived extracellular matrix and titanium: Comparative kinome profile analysis
Source: J Cell Physiol. 2018 Jul 30;234(3):2984–96. doi: 10.1002/jcp.27116 (PMC6585805; doi:10.1002/jcp.27116)

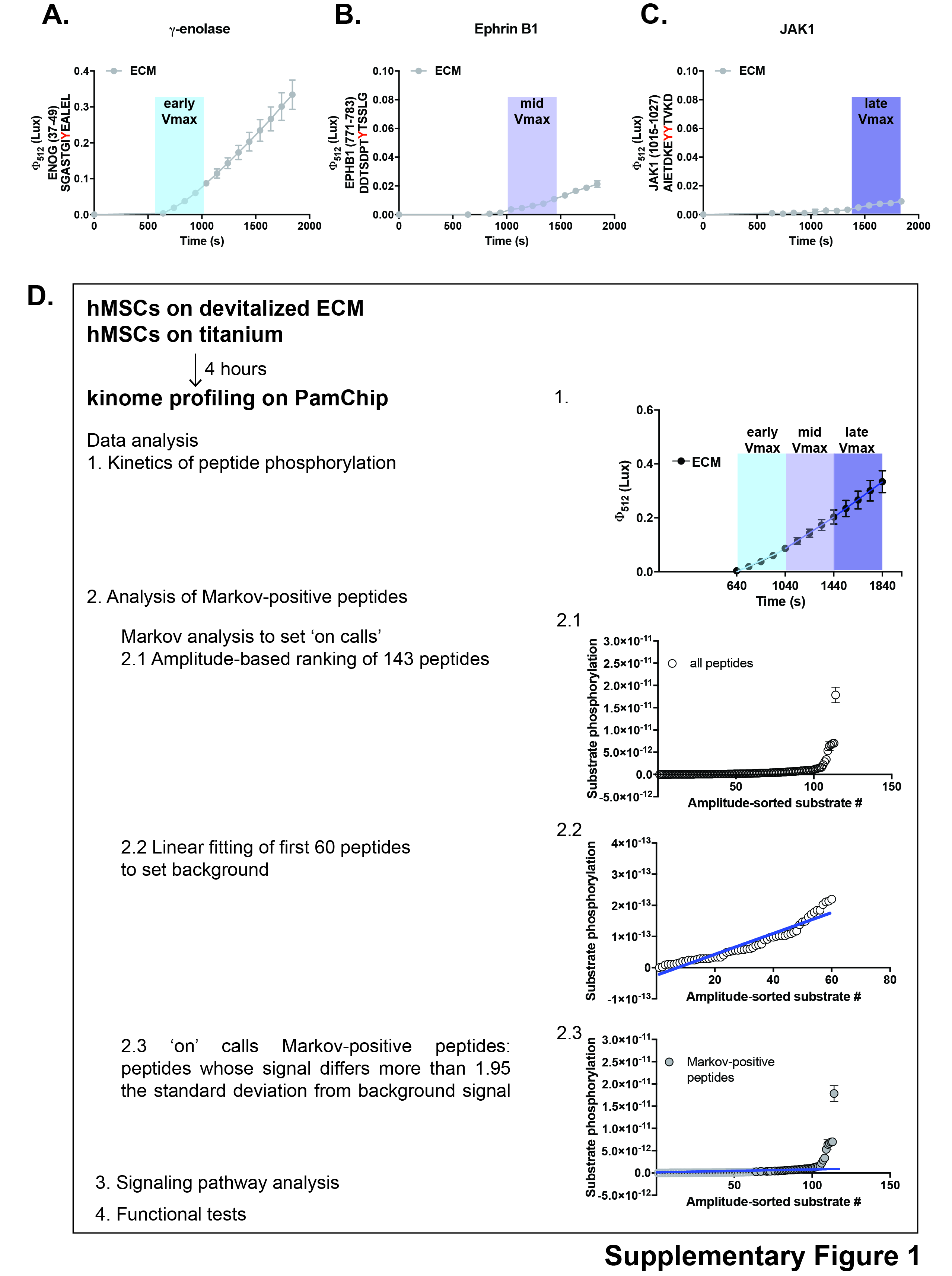

Supplement: Supplementary file 1 — Supporting information [file JCP-234-2984-s001.tif]

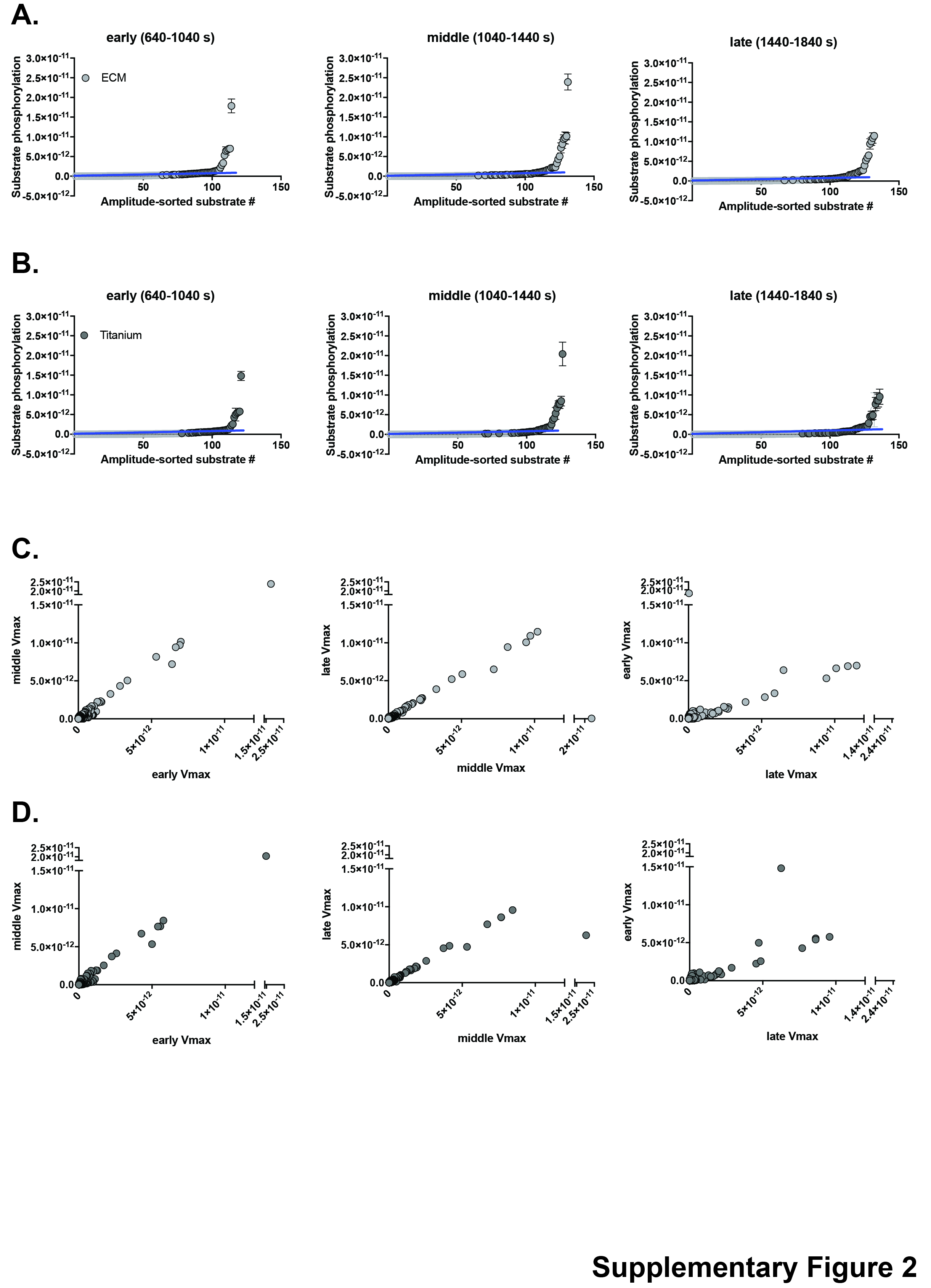

Supplement: Supplementary file 2 — Supporting information [file JCP-234-2984-s002.tif]

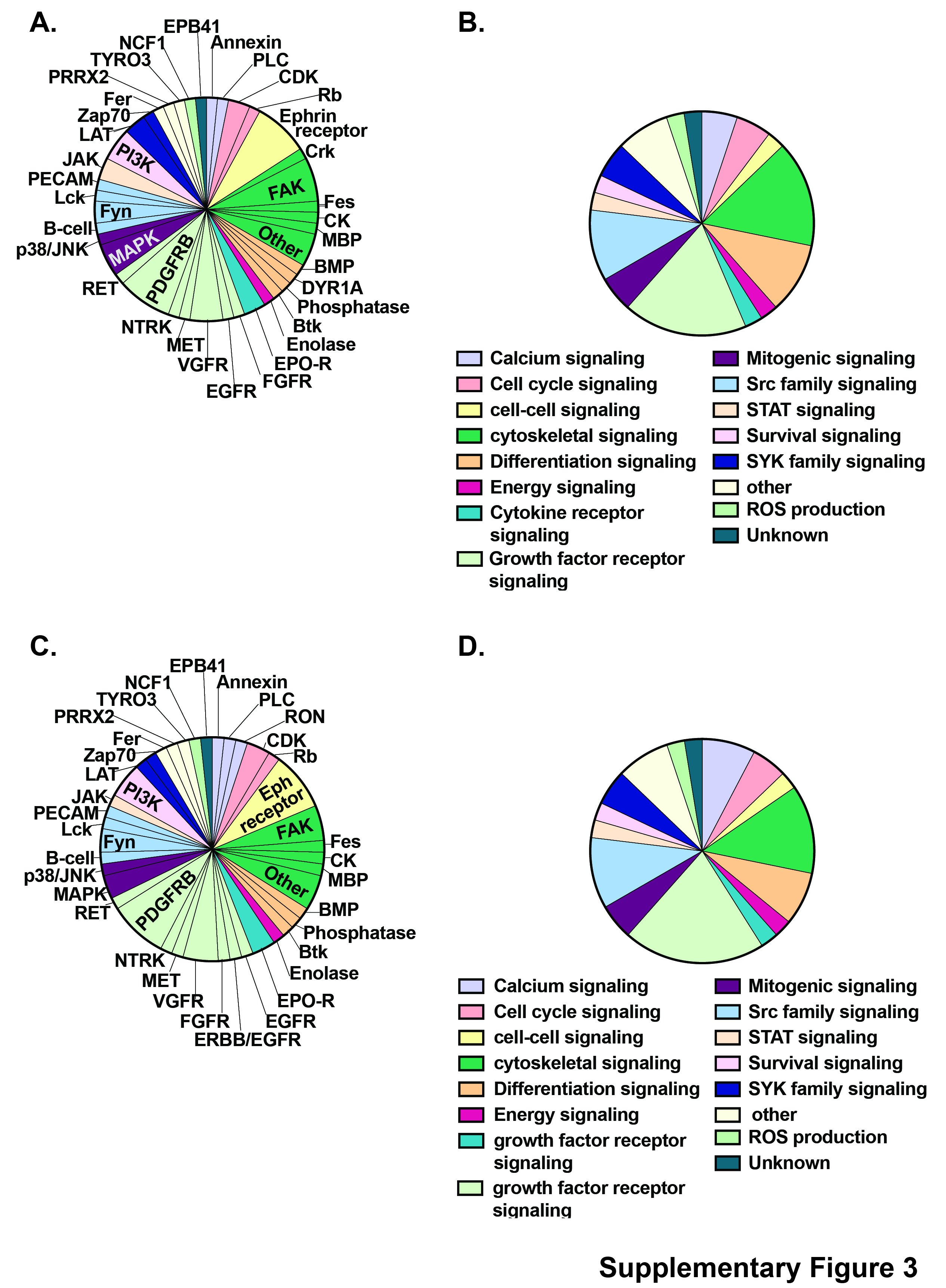

Supplement: Supplementary file 3 — Supporting information [file JCP-234-2984-s003.tif]

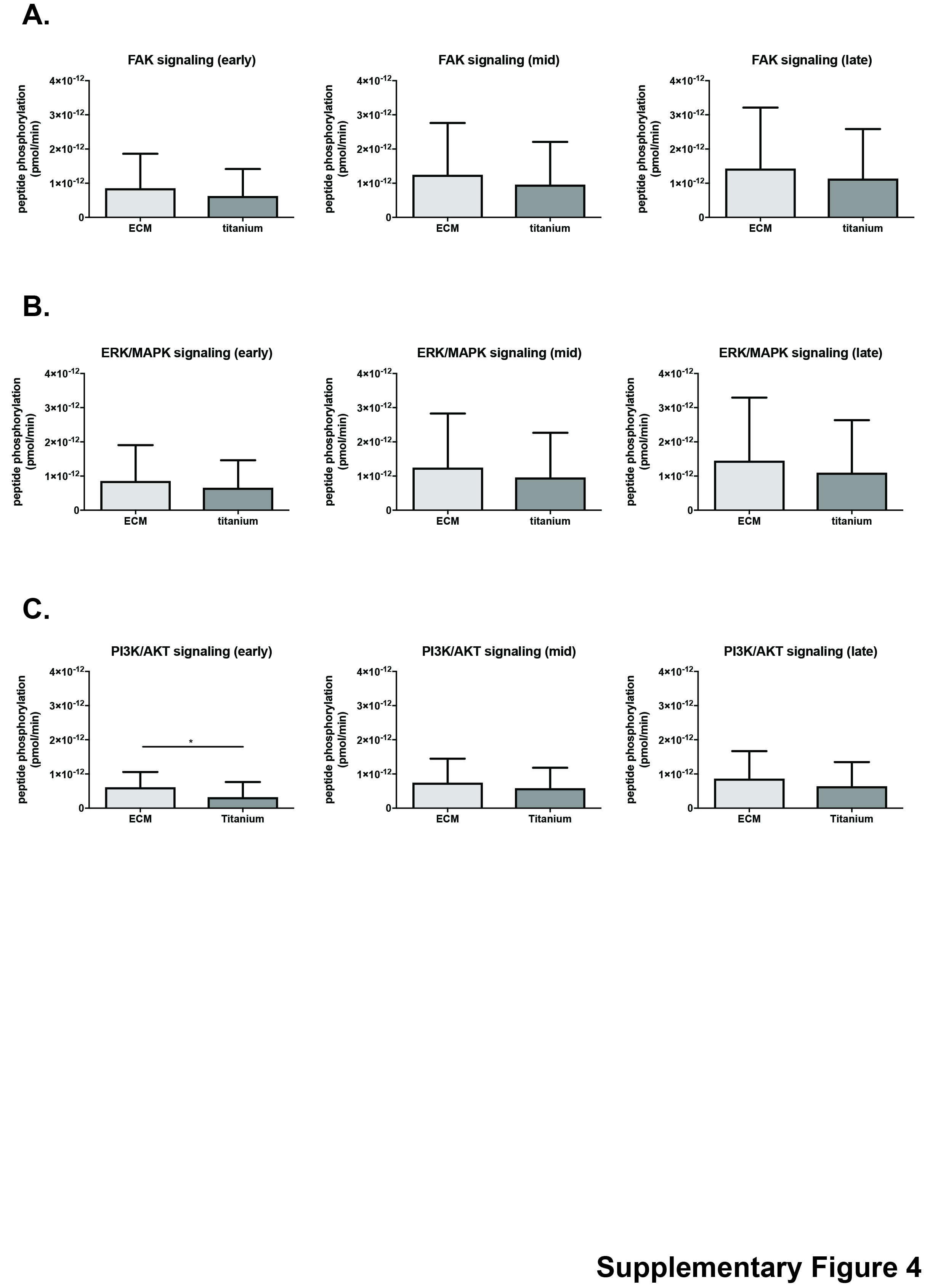

Supplement: Supplementary file 4 — Supporting information [file JCP-234-2984-s004.tif]

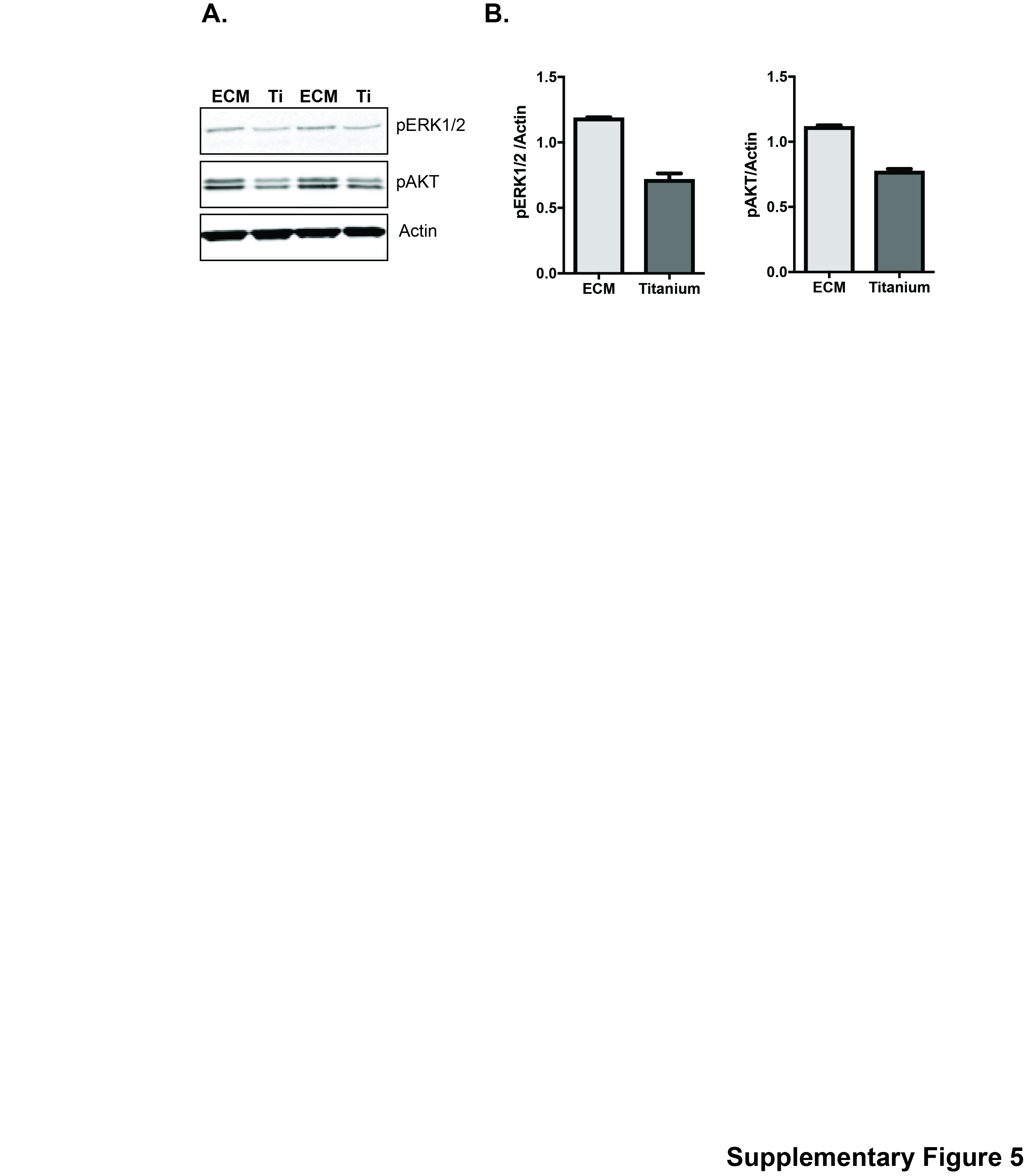

Supplement: Supplementary file 5 — Supporting information [file JCP-234-2984-s005.tif]
